# Unique associations of pain frequency and pain-related worry with health-related quality of life in survivors of childhood cancer

**DOI:** 10.1097/PR9.0000000000001000

**Published:** 2022-04-05

**Authors:** Lauren C. Heathcote, Sarah J. Cunningham, Michaela Patton, Fiona Schulte

**Affiliations:** a Health Psychology Section, Psychology Department, Institute of Psychiatry, Psychology and Neuroscience, King's College London, London, United Kingdom; b Department of Anesthesiology, Perioperative, and Pain Medicine, Stanford University School of Medicine, Stanford, CA, USA; Departments of c Psychology and; d Oncology, University of Calgary, Calgary, AB, Canada; e Division of Psychosocial Oncology, Alberta Children's Hospital, Calgary, AB, Canada

**Keywords:** Childhood cancer survivors, Cancer, Quality of life, Fear of cancer recurrence, Pain

## Abstract

**Introduction:**

Pain is common during childhood cancer treatment, can persist into survivorship, and can negatively affect health-related quality of life in survivors of childhood cancers (SCCs).

**Objective:**

The objective of this brief report was to assess pain frequency, pain-related worry, and their (unique) associations with health-related quality of life in SCCs.

**Methods:**

One hundred eleven SCCs (52% female individuals, *M* age: 17.67 years, range 8–25 years) completed self-report measures of pain frequency, pain-related worry, and health-related quality of life.

**Results:**

More than two-thirds (70%) of SCCs reported pain in the previous month (M = 1.39, SD = 1.17), and 15% reported experiencing pain often or almost always. More than one-third (39%) reported worrying about pain as a sign of cancer recurrence (M = 0.73, SD = 1.07), and 9% reported worrying about pain a lot or a whole lot. In multivariate regression models that controlled for sex, age at diagnosis, and time off treatment, both pain frequency and pain-related worry were significantly associated with physical health-related quality of life, indicating that they contribute unique variance to health-related quality of life after childhood cancer. For emotional health-related quality of life, pain frequency was no longer a significant predictor once pain-related worry was added to the model, indicating that pain-related worry may be particularly important for understanding emotional health-related quality of life.

**Conclusion:**

Postcancer pain may contribute to health-related quality of life through multiple mechanisms, including by triggering concerns of recurrence. There is a need for clinical interventions that target both the frequency of pain (eg, behavioral interventions) and pain-related worry (eg, psychoeducation and cognitive interventions) to improve health-related quality of life after childhood cancer.

## Abstract 2

More than one-third of childhood cancer survivors are worried about pain as a sign of disease recurrence; pain-related worry explained unique variance in health-related quality of life.

## 1. Introduction

Because survival rates for childhood cancers have now surpassed 80% and continue to rise, there is an increasing need to assess and address quality-of-life outcomes posttreatment.^10,11^ Pain is a central health-related quality-of-life (HRQoL) concern. Most studies that have aimed to characterize pain in survivors of childhood cancers (SCCs) have focused on long-term adult survivors.^9,11^ In youth SCCs, we know very little about the prevalence and nature of pain or how pain relates to HRQoL. Pain may affect HRQoL in SCCs through multiple pathways. Persistent, frequent pain may affect daily emotional and physical functioning, thereby reducing HRQoL. Recent qualitative studies suggest that everyday experiences of pain can trigger fears of recurrence years after cancer treatment.^1,3^ Thus, pain may indirectly affect HRQoL by maintaining fear of cancer recurrence, a primary HRQoL concern for cancer survivors.^12,14^

We aimed to characterize pain frequency and pain-related worry in a sample of SCCs in Canada. We predicted that survivors who experienced more frequent pain and greater pain-related worry would report a poorer HRQoL. Moreover, we predicted that pain frequency and pain-related worry would each account for unique variance in HRQoL, thereby indicating unique associations with HRQoL that may indicate distinct mechanisms. These findings can help guide intervention targets for comprehensively addressing pain after childhood cancer.

## 2. Methods

### 2.1. Participants

Participants were recruited from the Hematology, Oncology, and Transplant Program at Alberta Children's Hospital (91%) or through online social platforms (9%). Survivors (older than 18 years) and caregivers (younger than 18 years) provided consent. Eligible participants were (1) in the age group of 8 to 25 years, (2) diagnosed with cancer before the age of 21 years, (3) at least 2 years posttreatment or had finished treatment and were 5 years postdiagnosis, (4) living in Canada, and (5) fluent in English. Exclusion criteria were as follows: (1) developmental disabilities, (2) major injuries, surgeries, or illnesses within the previous year, and (3) a history of psychosis. Data from this sample have been reported elsewhere^1^; this brief report is the first to focus on pain outcomes.

### 2.2. Measures

#### 2.2.1. Sociodemographic and clinical characteristics

Sex, diagnosis, and treatment information were extracted from the child's medical records. The Intensity of Treatment scale^5^ was used to categorize treatment intensity based on the previous diagnosis, stage of disease, and treatment information.^1^ Caregivers reported their child's age, race, age at diagnosis, and time off treatment.

#### 2.2.2. Pain frequency and worry

The Symptom Worry scale^1^ comprises 2 subscales assessing the extent to which respondents worry about 10 different somatic sensations as a sign of cancer recurrence (worry subscale) and how frequently they experienced those sensations over the previous month (frequency subscale). Both subscales are answered on a 5-point Likert scale from not at all to a whole lot (worry subscale) or never to almost always (frequency subscale). Pain was the first item in each subscale; only data from the pain items were used in this study.

#### 2.2.3. Health-related quality of life

The Pediatric Quality of Life Inventory 4.0 Generic Core Scales were used to assess HRQoL, including 4 separate subscales of physical, emotional, social, and school functioning. We examined each subscale separately to assess differential associations with pain frequency and pain-related worry. Survivors of childhood cancers completed versions that were validated for their age group.^15,2^ Items were reverse scored and linearly transformed to a 0 to 100 scale, with higher mean scores indicating a better HRQoL.

### 2.3. Data analysis

Bivariate Pearson correlations and *t* tests were performed to assess the relationship between demographic factors, medical factors (age at diagnosis, time off treatment, and treatment intensity), pain frequency, pain-related worry, and HRQoL domains. Hierarchical linear regression analyses were performed to examine the unique associations between pain frequency, worry, and HRQoL domains. Specifically, demographic and medical characteristics correlated with any of the HRQoL domains were entered into the first step, followed by pain frequency and pain-related worry. All analyses were performed using SPSS software, version 26.^4^ Data are available on request from the corresponding author.

## 3. Results

### 3.1. Sample characteristics

One hundred eleven childhood cancer survivors (52% female individuals, M age: 17.67 years, range 8–25 years) were included in this study. Participants completed treatment 2.84 to 21.75 years previously (M = 10.26 years, SD = 4.91) and had received a range of previous diagnoses (solid tumors: 48%, blood disorders: 22%, lymphoma: 19%, and CNS tumors: 11%) and treatments (chemotherapy: 99%, surgery: 71%, radiation: 38%, transplant, 16%). Survivors of childhood cancers were White (81%), Asian (7%), other/mixed ethnicity (6%), Arab (2%), Latin American (2%), and Indigenous Peoples of Canada (1%).

### 3.2. Unique associations of pain frequency and pain-related worry with health-related quality of life

More than two-thirds (70%) of participants reported pain in the previous month (M = 1.39, SD = 1.17), and 15% reported experiencing pain often or almost always. More than one-third (39%) reported worrying about pain as a sign of cancer recurrence (M = 0.73, SD = 1.07), and 9% reported worrying about pain a lot or a whole lot. Those who endorsed worrying about pain were 95% more likely to endorse experiencing pain in the previous month, χ^2^(1, N = 110) = 21.13, *P* < 0.001 (Pearson correlation: *r* = 0.46, *P* < 0.001). As summarized in Table 1, more frequent pain and greater pain-related worry were associated with worse physical and emotional HRQoL; thus, regression models were performed for both these HRQoL domains. More frequent pain, but not pain-related worry, was associated with worse social and school HRQoL. Older age at diagnosis was associated with worse physical HRQoL, whereasless time off treatment was associated with worse emotional and school HRQoL. Girls reported significantly poorer physical, emotional, and school, but not social, HRQoL. Treatment intensity, age, and race were not significantly associated with any HRQoL domains and thus were not included in the regression model. In the regression models (Table 2), pain frequency was a significant predictor of physical and emotional HRQoL at step 2. For physical HRQoL, both pain frequency and pain-related worry were significant predictors at step 3, indicating that they contribute unique variance to physical HRQoL. For emotional HRQoL, pain frequency was no longer a significant predictor once pain-related worry was added to the model, indicating that pain-related worry may be particularly important for understanding emotional HRQoL.

**Table 1:** The Pearson correlations and *t* tests.

HRQoL domain	Pain-related worry (*r*)	Pain frequency (*r*)	Age (*r*)	Sex (*t*)	Race (*t*)	Age at Dx (*r*)	Time off Tx (*r*)	Tx intensity (*r*)
Physical	−0.47***	−0.58***	−0.14	4.17	1.45	−0.27**	0.13	−0.08
Emotional	−0.33***	−0.34***	0.06	4.11	1.19	−0.15	0.22*	0.04
Social	−0.09	−0.23*	0.06	1.45	−0.32	−0.11	0.18	0.05
School	−0.12	−0.29**	0.05	2.35	1.51	−0.18	0.24*	0.16

Given the heterogeneity in our sample, race is categorized as a dichotomous variable (White, not White).

*P < 0.05, **P < 0.01, ***P < 0.001.

HRQoL, health-related quality of life.

**Table 2:** Hierarchical linear regression models for physical and emotional health-related quality of life.

	Physical HRQoL			Emotional HRQoL			Df
	Β	*R* ^2^	F	β	*R* ^2^	F	
Step 1		0.19	7.62***		0.18	6.56***	3.92
Time off treatment	−0.08			0.12			
Age at diagnosis	−0.29**			−0.07			
Sex	−0.35***			−0.36***			
Step 2		0.40	15.38***		0.22	6.52***	4.91
Time off treatment	0.03			0.17			
Age at diagnosis	−0.12			0.01			
Sex	−0.24**			−0.31**			
Pain frequency	−0.49***			−0.23*			
Step 3		0.46	15.23***		0.26	6.45***	5.90
Time off treatment	0.04			0.18			
Age at diagnosis	−0.10			0.03			
Sex	−0.22**			−0.29**			
Pain frequency	−0.37***			−0.13			
Pain worry	−0.27**			−0.23*			

*P < 0.05, **P < 0.01, ***P < 0.001.

HRQoL, health-related quality of life.

## 4. Discussion

Previous studies have reported pain prevalence rates in survivors of childhood cancers ranging from 4.3% to 75%.^9,11^ Our finding of 70% is a liberal estimate of those who reported some degree of pain in the previous month. We also found that 15% of participants experienced pain frequently (ie, often or almost always) over the previous month, pointing towards those for whom pain may be more problematic and impactful. This study is one of the few to report the prevalence of pain in youth childhood cancer survivors. In 2 previous studies, 47% of youth SCCs reported pain during at least 1 clinic visit^8^ and 26% reported chronic pain that had lasted 3 months or longer.^7^ Survivors of childhood cancers who experience persistent and frequent pain are likely those who have the greatest clinical need for pain relief and pain management.

More than one-third (39%) of participants reported worrying about pain as a sign of cancer recurrence. It was previously reported that 21% attribute their pain to cancer and its treatment,^6^ yet few have considered how childhood cancer survivors interpret their pain within the context of survivorship and ongoing risk of disease recurrence. In 2 qualitative studies, SCCs described worrying about pain in the previous tumor site, as well as everyday aches and pains as signs of cancer recurrence (despite no subsequent evidence of disease recurrence).^3,13^ In a previous report, bodily symptoms triggered fears of recurrence in 62% of young survivors, sometimes many years after finishing cancer treatment.^1^ Because pain is emerging as an especially worrisome symptom, and given growing evidence that a sizable number of childhood cancer survivors report persistent and frequent pain,^9,11^ it may be particularly important to address pain-related worries within childhood cancer survivorship care.

Aligning with our hypotheses, participants who reported more frequent pain and greater pain-related worry also reported poorer physical and emotional HRQoL. In comprehensive statistical models that controlled for sex, age at diagnosis, and time off treatment associated with HRQoL, pain frequency and pain-related worry each explained unique variance in physical HRQoL. Moreover, pain frequency no longer explained unique variance in emotional HRQoL once pain-related worry was added to the model. These findings indicate that pain may contribute to HRQoL through multiple, distinct mechanisms. Pain is an unpleasant sensory and emotional experience that can interrupt attention on everyday activities, interfere with achieving valued life goals, and lead to functional impairment, thereby reducing quality of life. Pain can also serve as a reminder of the previous cancer experience and trigger concerns of disease recurrence, thereby maintaining fear of cancer recurrence that negatively affects emotional functioning and quality of life.

This study has limitations, most notably that pain frequency was assessed within the preceding month and thus our data cannot speak to pain persistence. Pain-related worry was assessed with a single item, and we did not know which qualities or locations of pain were most worried about or whether worry centered around persistent pains or new acute pains. Moreover, the sample was predominantly White, which may explain the lack of observed differences for race in HRQoL. Nevertheless, the current findings provide novel evidence that postcancer pain may contribute to HRQoL through multiple mechanisms, indicating a need for clinical interventions that target both the frequency of pain (eg, behavioral interventions) and pain-related worry (eg, psychoeducation and cognitive interventions) to improve physical and emotional HRQoL after childhood cancer.

## Disclosures

The authors have no conflict of interest to declare.
